# The Chronic Health Effects of Work-Related Stressors Experienced by Police Communications Workers

**DOI:** 10.1016/j.shaw.2021.05.005

**Published:** 2021-05-21

**Authors:** Rodolfo A. Perez, Katelyn K. Jetelina, Jennifer M. Reingle Gonzalez

**Affiliations:** 1 Urban Institute, Justice Policy Center, Washington, DC, USA; 2 Department of Epidemiology, Human Genetics and Environmental Sciences, University of Texas School of Public Health in Dallas, TX, USA; 3 Meadows Mental Health Policy Institute, Dallas, TX, USA

**Keywords:** 911, Emergency communications, Health, Police, Qualitative

## Abstract

**Background:**

Law enforcement communications (i.e., 911 dispatch and call takers) is a challenging and stressful occupation. The purpose of this study is to identify the main stressors associated with employment in law enforcement communications, and to identify and provide context to how these stressors affect workers' health and wellbeing.

**Methods:**

This research study included focus groups with 23 call takers and 911 dispatchers employed by a large, urban law enforcement agency in 2018. Thematic analyses were conducted to identify trends.

**Results:**

Four themes of stressors emerged (i.e., the high stakes nature of some 911 calls for service, understaffing, supervisor-related stress, and recruiting practice). Two health-related themes emerged as being occupation-related: weight gain and poor sleep patterns/insufficient sleep). Specifically, participants reported negative eating habits resulting in weight gain and obesity, lack of sleep and irregular sleep schedules, and development of hypertension and/or diabetes since beginning their jobs.

**Conclusion:**

Law enforcement communications professionals experience a number of the same stressors facing law enforcement officers in patrol. These stressors, combined with the sedentary nature of the job, could result in long-term, chronic health problems.

## 1 Introduction

Law enforcement communications (i.e., 911 dispatch and call takers in emergency call centers) is a challenging and stressful occupation for workers. On a daily basis, law enforcement communications workers handle life-threatening events, interact with individuals in crisis, and must be able to determine appropriate responses within seconds [1]. Several aspects of the emergency call center environment are well documented contributors to stress. These stressors include perceived second-class citizenship compared to sworn law enforcement personnel, shift work, lack of breaks, poor financial compensation, confinement in substandard facilities, lack of support from supervisory staff, calls from abusive or irate citizens, and calls involving child abuse or child death, among others [2]. Data collected from dispatchers suggest that dispatchers who had higher levels of perceived stress also had elevated levels of cortisol [3]. Therefore, the stressors associated with the occupation have demonstrable, documented physiological effects on health.

Given the challenges facing emergency call center staff, it is not surprising that these stressors have detrimental effects on employee's physical and mental health. In a sample of 758,911 communications workers, 83% were either overweight or obese [4] in comparison to 6.6% of the U.S. population [5]. In this same study, communications personnel reported having an average of 17 health complaints per month [4]. Up to one-fourth of communications workers meet some criteria for post-traumatic stress disorder (PTSD), and 24% suffer from depressive symptoms [6]. However, the mechanistic pathways between stress and health among the workers is unclear. For example, previous literature shows higher levels of cortisol levels due to stress, obesity, physical complaints, and instances of depression and PTSD but does not detail how stress is connected to these outcomes [3,4,6]. Therefore, research that describes how stress and negative health outcomes are interrelated among communications workers is needed.

Although the previous studies provide valuable insights into the health implications of working in an emergency call center, the research on this population is otherwise sparse, quantitative in nature, and does not provide context into the problem. To address this gap, the purpose of this study was to not only identify the main stressors associated with employment in law enforcement communications, but to also identify how these stressors impact workers' health and wellbeing. As is common in qualitative research, we did not collect data with specific *a priori* hypotheses in mind [7]. However, we expected to detect negative coping strategies (e.g., substance use; specifically, alcohol consumption) and health behaviors (e.g., poor eating and sleeping patterns, insufficient exercise) that lead to adverse chronic health outcomes.

## 2 Materials and methods

### 2.1 Procedure

Three semi-structured focus groups were conducted with 23 communications employees, including primarily dispatchers and call takers, as well as three representatives from other departments (the city's National Crime Information Center division and reporting—both of which are classified as “communications” and are co-located with the 911 call center). These professionals work directly with the public to answer 911 calls for police service; and dispatchers, who liaise between call takers and police officers to assign officers to respond to the call for service. All communications personnel were civilians employed by a large urban police department in the United States.

Sworn law enforcement personnel outside of each participant's chain of command contacted all communications workers who were expected to report for work on each of the predetermined data collection days. Prospective participants were notified that 1-hour focus groups for a “health study” were being conducted on-site, and that anyone who wishes to participate should present 1 hour before or after their shift to the location. Interested individuals signed up for a focus group session scheduled to occur either before or after their shift. Focus groups were conducted before or following each of the three shifts (morning or day shift, evening shift, and night shift). At the beginning of the focus group, participants received information about this voluntary study, and they were informed that their employment was not contingent upon study participation.

A total of 23 communications workers participated in focus groups. Demographic data were not collected to protect participant anonymity given the sensitive nature of the topics. An assessment of participant demographics by the researcher who led the interviews identified nearly all participants as women and more than half were African American or Black. The moderator for all three focus groups was the principal investigator of the study (author JMRG). This author has 10 years of experience in conducting qualitative and mixed-methods research. Specifically, she has led all qualitative data collation methodological design, collection, and analysis for two large National Institutes of Health funded research projects. She was also the principal investigator of two funded projects that included a substantial amount of qualitative data. These projects have resulted in seven peer-reviewed publications [[8], [9], [10], [11], [12], [13], [14]] and one report that includes exclusively qualitative data [15]. The principal investigator has also co-authored a research methods textbook that includes a chapter solely focused on qualitative and mixed-methods data collection methodologies [16].

During the focus groups, the moderator asked participants to describe the main stressors associated with their position and how these stressors affect their health and wellbeing. All focus groups were audio recorded. Focus group lasted approximately 1 hour and a $30 gift card was provided to participants in exchange for their time. Specific questions are available in the appendix. The Committee for the Protection of Human Subjects at the University of [Blinded] approved the data collection protocol for this project (IRB Approval # Blinded).

### 2.2 Data coding and analysis

Systematic procedures of qualitative data analysis included intensive, repetitive review of audio recordings, discussion of the transcripts by the research team, coding by two investigators (the PI and trained research staff), inductive thematic identification, data reduction, and interpretation. The research staff member charged with initial coding of the data recorded key excepts from participants, and the number of times that each theme emerged in the discussion. Inconsistencies in the coding process and results were resolved by the research team. Given the limited number of focus groups conducted, no specialized software was used for data coding or analysis.

## 3 Results

Across the three focus groups, four themes of stressors emerged (i.e., nature of call, understaffing, supervisor training, and recruiting practice) and two health-related themes emerged (weight gain and sleep). Each of these domains of themes is described in detail below.

### 3.1 Stressors

Nature of calls. Communications workers consistently reported the emotional nature of 911 callers. As a 911 call taker stated,

“You can get someone who is calling in and it's a disturbance call and [the civilian is] screaming and yelling because they're upset … you have to remain calm. You can't feed into their frustration. You go to make sure that you ask all of the questions that are needed. You have to do your due diligence to take care of the officers in the field to make sure that they're just not walking into something blindly.”

Injury and/or mortality was particularly difficult for call takers, “If I just had someone to get shot or [a] child dying, even though we've done it a while, you're still human”, or “To me what's stressful is when you're speaking with someone and they're either shot or cut, or injured or something. That gets to be stressful for me because then I have the fear of them dying or something while I'm talking to them on the phone.” As broadly stated by one call taker, “You never know what's going to come through. And regardless to how nice you're trying to be, how customer service oriented, it's still horrible.”

#### 3.1.1 Understaffing

Communications employees reported understaffing at the emergency call center as a consistent issue that caused stress. Turnover was identified as a primary cause of this constant understaffing. As stated by one participant, “People come in, they don't stay. Period. Some of them don't even make it through that training session.” As a result, the remaining employees must work overtime to ensure sufficient manpower. Another participant noted, “It's stressful because all of us that are here, we end up having to work double and triple shifts to make up for that lost body.” Participants noted that they are often unable to get days off because of minimum staffing requirements. In summary, based on the focus group findings, staff turnover was the primary driver of understaffing. The understaffing created by staff turnover created other issues such as having to work overtime and the inability to take days off due to minimum staffing requirements.

Turnover is driven largely by low pay, “When you start paying your officers [more money], we'll start getting more officers in. We can't compete [with suburban agencies]. We don't compete with other cities because the money is just not there … And with those other cities, our call volume is double or triple. So, why would [a potential recruit] come here?”

Because of turnover, communications workers are often logging long work shifts totaling up to sixteen hours at a time. Although this overtime is not technically mandatory, workers mentioned that they “want [ed] to pick up the overtime to avoid being mandated.” As one dispatcher stated,

“We want to have time to work out and to take care of ourselves but … I would rather work the over time than have somebody here who is not doing their job and is going to get somebody killed because we develop relationships with our officers and we care about our officers. We don't want them hurt. I would much rather work three 16-hour days in a row like I am doing this week to make sure that it’s staffed.”

Understaffing of patrol officers adds stress on communication workers. As one 911 call taker stated, “I know I get a lot of angry customers (e.g., civilians contacting 911) because they've been waiting on officers forever. And it adds stress to us [because] when we hear stressfulness in their voices saying, ‘Where's the police? I need the police. I'm trying to get this man out of my house. Where are the officers? I called 3 or 4 hours ago.’ So that adds burden, stress, and anxiety for us.”

#### 3.1.2 Supervisor training

The limited number of law enforcement officers in the department has also resulted in the civilianization of communications functions, and the communications rank and file perceive that these civilians (particularly the supervisors) are not as familiar with the organizational structure and purpose when compared to sworn personnel. Participants reported that civilian staff are also not trained in management as thoroughly as sworn officers, and civilian managers place a lot of pressure and demands on staff because of their unfamiliarity with police stress. For example, several participants noted that an error does not result in professional coaching; rather, civilian supervisors often generate memoranda that must be signed and placed in an employee's personnel file. This created a sense of frustration,

“the person that usually does something wrong half the time doesn't realize that they [did] something wrong. So they go about their business while the rest of us are ticked off [for having to sign the memo]”, and “You're stepping on those who are doing their jobs right instead of going after those that need to be educated and trained or re-trained or whatever the case may be.”

Several participants also mentioned that civilian managers are well-known for micromanaging and demeaning behavior, which can lead to turnover and low morale. As stated by one 911 call taker, “It's hard to listen to your callers and assist the callers when [the managers are] yelling. Especially when [the civilian managers] are yelling out things that are demeaning like, ‘We're going to take your lunch time away if our service levels don't go up!’ And that is a huge stressor as an adult when you're on the phone taking a stressful call and you have the management team yelling at you. So, you're trying to listen to both parties. You have your caller that you need to focus on yet you got your management team yelling something.”

#### 3.1.3 Recruiting practices

Results from our focus groups suggest that standards for new hires appear to have declined over time, and that hiring standards need to be raised. As one communications worker stated, “[Leadership is] in a hurry to staff [the call center] and I think they lower some of their standards and they should not.” These hires make the job more stressful because others have to compensate for the underqualified employees. Participants suggested that quality is more important than quantity in terms of new hires,

“I can teach anybody to take a 911 call. It doesn't mean you're going to be great at it. It doesn't mean it's your calling. I can teach anybody off the street to take a 911 call. That doesn't mean that's what you're meant to do. I mean, if you get in and you find out this person can't do it or they're just not meant for this profession, get them out of here. None of us need that stress. Because it stresses everybody else that does love their job.”

As a result, “we're having to pick up the slack from somebody else [who does not love their job].”

### 3.2 Work stressors that impact health

Nearly all participants asserted that their work has had a detrimental effect on their health. Because lunch breaks are sometimes restricted or eliminated, participants reported binge eating after their shift:

“With all the stress you just eat … You don't eat [at work] and then at the end of your shift you're cleaning out the fridge because you didn't get a chance to eat or snack through your shift. Or you only had a little snack and then you get off work and you're like, ‘You know, I haven't eaten in 10 hours let me go eat half my refrigerator.’”

Similarly, one 911 call taker stated that she managed her stress by eating unhealthy food, “You get stressed [at work] so the first thing you do is run to the vending machine.” Condensed break times and lack of personal time to recharge were highlighted by another call center employee:

“When we take lunch, we warm our [meal] up in the microwave, roll over to the table and eat, and roll back to the [call center] desk because there is always something going. So, we don't even get the opportunity to walk out somewhere for 30 minutes.”

Although a kitchen is available to employees, it is not widely used because breaks are brief. One dispatcher stated explicitly, “It definitely takes a really concentrated effort to eat well on this type of job.” As a result, employees reported weight gain, diagnoses of hypertension, thyroid problems, and diabetes after beginning their jobs. For example, “When I started this job, I've only been here 2 years in October. And since I've been here I gained 25 pounds”; “I gained weight and I developed hypertension”; and, “I never has a blood pressure problem before I came here.” All city employees have access to an exercise facility that they can use free of charge on their breaks, or before or after shifts. Participants stated that there is not sufficient time to use the workout room because their breaks were 30 minutes or less: “I used to exercise all the time. Especially when we had the hours breaks because we had time go to over to the wellness center.” As a result, weight gain in excess of 25 pounds was noted by multiple participants.

## 4 Discussion

This study identified a number of stressors, including pressure to deal with life and death situations properly and rapidly, call taker and dispatcher perceived high stakes of the job, understaffing, and high workloads often resulting in long shifts with few breaks and frequent overtime. Nearly all participants asserted that their work has had a detrimental effect on their health. Specifically, participants reported negative eating habits resulting in weight gain, obesity, and obesity—related disorders (e.g., hypertension and/or diabetes) since beginning their jobs.

The stressors identified in this study are consistent with those identified in prior studies. Conflict between communications workers and supervisory staff has been previously documented [2], and results from the present study suggest that communications workers are frequently in conflict with their supervisors. Our study's participants perceived that these supervisors were not well trained or qualified to manage, and this promoted stress among participants. The call taker and dispatcher roles are unique from other professions in that workers need to be able to successfully and tactfully communicate with more than one person at the same time. For example, dispatchers are communicating with call takers and officers (and often, other contractors such as tow truck companies), while call takers must juggle an emergency call from a civilian with dispatcher communication. When supervisors interfere with this process, they add an additional layer of complexity and stress to communication workers' jobs.

In addition, call takers reported that abusive behavior by callers in crisis is a critical stressor, and this is supported by extant research. Specifically, Bendini and colleagues [3] found that salivary cortisol levels were highest when workers were answering incoming emergency calls compared to when the same workers were working dispatch functions. Therefore, it appears that the call taker position may be inherently stressful, and these workers may need more breaks and gaps between calls to decompress and manage their stress to avoid burnout. Instead, results from this study suggest that breaks are brief (when they are available), and some supervisors may threaten to shorten or eliminate breaks as a punishment for lack of productivity (e.g., too many calls are holding).

The health consequences associated with communications workers have been less studied. The present study identified specific stressors that appeared to directly impact obesity and weight gain, and these findings are consistent with past literature. According to one national survey of communications workers, only 18% reported a body mass index within the normal weight range [4]. Extant research shows that physical health can be improved by addressing mental health [4], which may provide an opportunity for peers or supervisors to identify and refer communications workers suffering from mental health symptomology to needed services, with the long-term goal of improving the physical health of communications workers. Longitudinal studies are needed to document the development of physical health and mental health problems among communications workers with the goal of evaluating temporal ambiguity, and ultimately improving their holistic wellbeing.

Finally, communication worker job dissatisfaction, including perceived low pay and insufficient promotional opportunities, has been linked to burnout and stress on the job [2]. The perception of low status has also been identified in the literature [2], and this was discussed by communications workers in the present study. These factors may be at least partially addressed through performance recognition, salary increases and non-monetary rewards (e.g., days off or early dismissal for performance, public recognition) [1]. In 2019, the Texas State Legislature expanded the definition of ‘first responder’ to include 911 telecommunicators (or, communications workers) [17]. This classification change may permit jurisdictions to compensate communications workers for certifications earned, thus providing an opportunity to enhance wages in this population.

Notably, mental health was not identified as a theme in our focus groups, and this may be a function of the stigma associated with self-reporting mental health conditions in a public forum. Other studies on the mental health of communications workers have found that the prevalence of depression (24%) exceeds the rates reported in samples of law enforcement officers [6,18,19], and duty-related experiences were significantly associated with greater mental health concerns [4]. These high rates of stress-related mental health disorders identified in the literature are not surprising given the magnitude of the physical health impacts of stress facing communications workers in our study.

### 4.1 Strengths & limitations

The results from the present study should be considered in light of its limitations. First, the sample was relatively small and included only 23 communications workers from a single, urban department. This pilot study was conducted as part of a larger project and likely did not capture themes to saturation. In light of these weaknesses, our study was diverse across communications subspecialties (e.g., call takers and dispatchers, as well smaller divisions within communications) and shifts, which could have an impact on themes identified. Furthermore, our study is the first to provide context of how these stressors are linked to poor physical outcomes.

### 4.2 Recommendations for policy future research

Findings from this study and the totality of the literature suggest a number of opportunities for policy and practice modifications to reduce stress and improve health in communications workers. Communications divisions should examine the use of internal promotional policies to reward experienced and highly qualified staff rather than hiring supervisors from the outside [2]. This may improve morale, perceived worth, and as a result, reduce stress. If funds are available, increases in salary and benefits have documented effects on reduced occupational strain [2], and additional personnel would allow for communications staff to work shorter shifts and accommodate needed time off from work. Communications divisions should also provide the necessary training and support for new personnel so that they are well prepared to assume the position and do not burden existing staff [2]. For example, the Caruth Police Institute (among other public safety training institutes) provides a civilian leadership training series to equip civilian supervisors with the tools they need to successfully manage personnel. This training should be disseminated more broadly to civilians in departments nationwide that may not have the resources needed to develop such a training program alone.

Future studies should further investigate these linkages by measuring specific somatic and mental health symptoms before an individual joins the communications profession and tracking these symptoms over time. Policy and practice evaluations of the modifications discussed earlier should also be conducted to examine whether these practices have an impact on communications occupational health.

## Conflicts of interest

The authors have no conflicts of interest to report.
