# Supplementary material for: The Chronic Health Effects of Work-Related Stressors Experienced by Police Communications Workers
Source: Saf Health Work. 2021 May 21;12(3):365–9. doi: 10.1016/j.shaw.2021.05.005 (PMC8430437; doi:10.1016/j.shaw.2021.05.005)
Supplement: Multimedia component 1 [file mmc1.pdf]

The Law Enforcement Officer Stress Surveillance Study (LEO-STRESS)

FOCUS GROUP SCRIPT- 911 Call Center

I am [redacted], a researcher at the [redacted]. I have been conducting a study with new [redacted] officers to measure their stress associated with the law enforcement occupation (project called LEO-STRESS). I would like to also get your feedback stress associated with your job at the 911 Call Center, and more specifically, your attitudes and use of the new Down Room. I think this session will last approximately one hour.

My ultimate goal is to better understand the impact of your routine activities on your health. This information will help my team inform a larger project and potential interventions that can help reduce negative effects of stress on your health. Since you have been participating in this project and you are the experts here, I need your perspective to provide context and background information to help me identify best practices and areas for future investigation.

The format of today's discussion is a semi-structured focus group. In a semi-structured focus group, I have a list of questions to ask, but I may also ask probing questions in order to better understand your answers. For example, "why do you say that?" or "let us explore this theme a little more. Therefore, please feel free to be completely open and honest today.

All of your responses will be kept confidential, and please respect others by not sharing their responses outside of this discussion. In order to protect your confidentiality, please do not use each others' names when responding to questions or addressing other participants. It is also important to give everyone an opportunity to speak. There are no right or wrong answers - I am interested in your opinions.

I will be recording today's discussion with a digital recorder, and the discussion will be transcribed and saved in a protected, private place. Your personal information will not appear in the transcribed notes. Your names will not be associated with the summary of this discussion. None of you will be asked to identify yourselves by name during the recording. I will destroy all electronic copies of this discussion in five years to maintain compliance with federal regulations.

Before I begin our discussion today, each of you should have received, read and signed a consent form stating that your participation in this discussion is completely voluntary and you are able to leave at any time for any reason. In exchange for your time today, you will receive a $30 gift card.

As a reminder, your answers and comments will remain confidential and any reports from this conversation will not specify your name or any other identifying information about you. Your individual responses will not be reported back your employer or the agency that funded this project. Your participation is voluntary and will not affect your employment status. If you feel uncomfortable with the recording, please let me know and you can leave before the process begins. You may also stop participating in the discussion at any time.

Are there any questions at this time about the consent form?

Do you agree to participate in this project?

Thank you for your time and participation. Let's begin the discussion.

[redacted]

Moderator: I want to know what you think. There are no right or wrong answers to the following questions, and all of your opinions are valuable.

Stress Items

1. In general, 911 dispatchers and call takers experience a disproportionate amount of chronic disease. Our goal is to better understand the causes of this disparity. What do you think is driving the high rates of disease?

2. In your opinion, what are your greatest stressors (work- and non-work related)?

   2a. What do you do to cope with these work- and non-work related stressors?

   2b. Does the timing of these events (e.g., early vs. late in your shift, day or night) impact your ability to manage the stress associated with these events?

   2c. Are there any activities or rituals you use to manage the stress?

3. Have your eating habits changed since you got in this line of work?

   3a. If yes, how?

Organizational Structure

4. The organizational structure of your job is unique in that you are civilians yet those on the other side of the radio are sworn officers. How does the citizen-officer dynamic affect your stress?

5. You also have long shifts. Has the newly implemented shift change affected your stress or your job?

   5a. How has this affected your morale?

[redacted]

Down Room

6. Have you utilized the Down Room?

   a. If yes, tell us about your experience.

   b. Were there specific calls or situations that this room was most helpful?

7. Does the Down Room help reduce work related stress and anxiety?

8. Was there ever a time you wanted to utilize the Down Room but were not able?

   8a. If yes, please describe your experience.

Implications

9. Our team is committed to improving the health of people who work in stressful occupations. What other kinds of programs or policies could the department implement to help reduce your stress levels?

10. What kinds of programs or policies could the department implement to improve your health?

Moderator: Is there anything we missed that you would like to talk about?

Moderator: I really appreciate all of your participation in this study. Thank you for your time.

[redacted]
